# The prevalence and determinants of polypharmacy at age 69: a British birth cohort study

**DOI:** 10.1186/s12877-018-0795-2

**Published:** 2018-05-16

**Authors:** Mark James Rawle, Marcus Richards, Daniel Davis, Diana Kuh

**Affiliations:** Medical Research Council Unit for Lifelong Health and Ageing at University College London, 33 Bedford Place, London, WC1B 5JU England

**Keywords:** Polypharmacy, Education, Social class, Socioeconomic position, Epidemiology, Disease burden, Cardiology, Cohort, Life course

## Abstract

**Background:**

To describe the development of polypharmacy and its components in a British birth cohort in its seventh decade and to investigate socioeconomic and gender differences independent of disease burden.

**Methods:**

Data from the MRC National Survey for Health and Development were analysed to determine the prevalence and composition of polypharmacy at age 69 and changes since ages 60 to 64. Multinomial regression was used to test associations between gender, education and occupational social class and total, cardiological and non-cardiological polypharmacy controlling for disease burden.

**Results:**

At age 69, 22.8% of individuals were taking more than 5 medications. There was an increase in the use of 5 to 8 medications (+ 2.3%) and over 9 medications (+ 0.8%) between ages 60–64 and 69. The greatest increases were found for cardiovascular (+ 13.4%) and gastrointestinal medications (+ 7.3%). Men experienced greater cardiological polypharmacy, women greater non-cardiological polypharmacy. Higher levels of education were associated with lower polypharmacy independent of disease burden, with strongest effects seen for over five cardiological medications (RRR 0.3, 95% CI 0.2–0.5 *p* < 0.001 for advanced secondary qualifications compared with no qualification); there was no additional effect of social class.

**Conclusions:**

Polypharmacy increased over the seventh decade. Those with lower levels of education had more polypharmacy (total, cardiological and non-cardiological), even allowing for disease burden. Further analysis of future outcomes resulting from polypharmacy should take into account educational and gender differences, in an effort to identify at-risk populations who could benefit from medication reviews.

## Background

Polypharmacy is growing in the general population, with over half of those over age 65 in the UK taking more than three prescribed medications [1]. Between 1995 and 2010 the proportion of individuals prescribed five or more medications in a large-scale Scottish cohort study rose from 11.4 to 20.8% of all adults [2]. As individual lifespans increase, more chronic diseases accumulate over time; and a growing catalogue of pharmacological treatments for these diseases results in higher numbers of prescriptions [3]. In the MRC National Survey for Health and Development (NSHD), we previously reported that by their early sixties the majority of men and women have at least two clinical disorders requiring monitoring or treatment [4]; and by their late sixties, one in five have three or more doctor-diagnosed disorders [5]. While an increase in therapeutic options for chronic disease is welcome, often the complex interplay between these medicines is less well accounted for. Adverse drug reactions are estimated to account for around 6.5% of all hospital admissions in the UK, the majority of which are avoidable [6]. The risk of a significant interaction between pharmaceutical therapies rises along with the number of prescribed medications [7]. It is for this reason that polypharmacy has been linked with increased risk of falls, reduced functional status and cognition, and higher all-cause mortality in later life [8–10].

Prior research has identified that female gender [11, 12], lower socioeconomic position and lower education [13, 14] increases the risk of polypharmacy. These groups are also at significantly increased risk of multi-morbidity, the key driver of polypharmacy [11, 15]. In a large scale analysis of Scottish primary care health records, those in more economically deprived areas had an earlier onset of multi-morbidity, and a higher proportion of chronic obstructive pulmonary disease (COPD), painful disorders and depression [16]. Those on treatments for specific disorders, such as cardiovascular syndromes, are far more likely to be on multiple therapeutic agents [3, 11], perhaps as a result of more established evidence bases for the use of multi-drug treatment regimens in this field. These drugs vary in their side-effects and risk of interaction with other pharmaceutical agents [17]. Cardiovascular medications in particular are most commonly associated with adverse drug reactions in older adults, and this is the most frequently prescribed British National Formulary (BNF) category of medication in the general population [2, 18]. Men, and individuals from lower socioeconomic groups, are more likely to suffer from cardiovascular disorders [4, 19] and consequently cardiovascular polypharmacy [20, 21].

Within studies on its determinants, polypharmacy is commonly defined by an arbitrary cut-off of greater than a set number of total medications [22, 23]. Given both the prominence of cardiovascular medication prescription in older adults, and the increased side effect profile they carry, it is worth distinguishing between cardiological and non-cardiological polypharmacy and investigating the effects of gender and socioeconomic variation, independent of disease burden, on composition of polypharmacy. This understanding of the genesis of cardiovascular polypharmacy might help us better identify at-risk groups who may benefit from targeted interventions to reduce potential harm caused by adverse drug events.

We use the oldest of the British birth cohort studies to: a) describe medication use, general polypharmacy, cardiological polypharmacy and non-cardiological polypharmacy at age 69; b) assess the change in medication use across the seventh decade of life; and c) investigate how gender, education and adult socioeconomic position are associated with these types of polypharmacy. We hypothesised that being more educationally or socioeconomically disadvantaged would be associated with polypharmacy even after taking account of disease burden.

## Methods

The MRC National Survey for Health and Development (NSHD), has followed 5362 individuals (2547 women) since their birth in England, Scotland & Wales in a single week of March 1946, so far to age 70 [5, 24]. The most recent data collection was conducted between 2014 and 2015, when study members were aged 68–69 years. Prior assessment of study member responses confirmed the NSHD as representative of this population at the age 60–64 [25]; since then, additional losses to follow-up other than death have remained very low [5]. At 68–69 years, following a postal questionnaire, study members still alive and with a known current address in mainland Britain (*n* = 2698) were invited to have a home visit at age 69; 2149 (79.7%) visits were completed. Invitations were not sent to those who had died (*n* = 995), who were living abroad (*n* = 583), who restrict participation to postal questionnaires (*n* = 22), had previously withdrawn from the study (*n* = 632) or had been lost to follow-up (*n* = 432) [5].

### Prescribed medications

Information on regularly prescribed medication was collected by the research nurse at age 69 and at the previous data collection at age 60–64 [24]. At both follow-ups, the nurse recorded all regularly prescribed medications, including ‘as required’ medication that was regularly used, preferably using written lists provided by the participant rather than relying on verbal recall. Medications were coded by brand name and then standardised to generic pharmaceutical names and grouped by BNF code and chapter [26]. Drug data within each BNF chapter were subdivided into subcategories of pharmaceutical agent based on mode of action, primarily dictated by BNF chapter subsection; with further separation of specific agents that are commonly co-prescribed, including anti-platelets, bronchodilators, antiepileptic medications, dopaminergic antiparkinsonian medications, insulin and oral antidiabetic agents. From these data we derived a total count of medications, and an indicator of general polypharmacy adapted from pre-existing thresholds [23], namely 5–8 medications (polypharmacy) and 9 or more medications (extreme polypharmacy). We also created a total count of cardiological medications (based on Chapter 2 of the BNF) and non-cardiological medications (all remaining medications), and classified these into four categories: no medications, one medication (monotherapy), 2–4 medications cardiological/non cardiological polypharmacy) 5 or more medications (extreme cardiological/non-cardiological polypharmacy).

### Explanatory variables

Explanatory variables chosen were: gender [11, 12]; highest educational qualifications achieved by age 26 [13], grouped into three categories (none; GCSE ordinary secondary level or their equivalents; advanced secondary-level or higher); own current or if missing previous occupation by age 53 [2], based on the Registrar General’s classification of own occupation using a dichotomous split between manual (III-M, IV & V) and non-manual (I, II & III-NM); and disease burden at age 69 [3, 12, 27]. Disease burden was defined by two measures. The first was a count of the number of doctor diagnosed chronic diseases or disorders over the last 10 years, reported by participants. The research nurse asked about 19 disorders: heart failure, angina, myocardial infarction, hyper/hypotension, stroke, diabetes, transient ischaemic attacks, cancer, chronic lung disease, asthma, osteoarthritis, rheumatoid arthritis, osteoporosis, serious eye trouble, depression, epilepsy, Parkinson’s disease, memory problems and kidney disease. On this scale, we distinguished individuals with 0, 1, 2 or 3 or more doctor diagnosed diseases. To additionally adjust for the severity of these diseases, as more severe illness may warrant more medications for treatment, the second measure was the participant’s binary yes/no response to the question “Do you have any long-term illness, health problem or disability that limits the activities/work you can do?”

### Statistical methods

Participant characteristics were described in terms of the distribution of explanatory variables, medication use at age 69, and the change in medication use from age 60–64. Multinomial regression models were used to investigate the relationships between the explanatory variables and extreme total polypharmacy, total polypharmacy, those taking fewer medications, and those taking no medication, with the latter as the reference group. The models were adjusted first for gender, then additionally for educational qualifications and adult social class (model one), and finally for disease burden (model two) to see whether this explained any observed socioeconomic differences. We repeated these models for cardiological and non-cardiological polypharmacy. All statistical analysis was conducted using Stata 14 (StataCorp, Texas).

## Results

There were 2122 (98.7% of participants, of whom 51% were female) with known medication data at age 69 (Table 1). Almost a third (31.2%) had no educational qualifications although almost three quarters had been in non-manual occupations. A fifth (20%) reported three or more doctor diagnosed diseases and another fifth (20.3%) reported two.

**Table 1:** Characteristics of 2122 MRC NSHD participants with medication data at age 69

Descriptive statistics	Age 69	
Sample Size	2122	
Female Gender	1084 (51.1%)	
Education Status	*2011*	
None	627 (31.2%)	
Vocational / O-Level	569 (28.3%)	
A-Level / Higher	815 (40.5%)	
Social Class	*2108*	
Manual Social Class	589 (27.9%)	
Non-Manual Social Class	1519 (72.1%)	
Number of Doctor Diagnosed Chronic Diseases	*2120*	
0	532 (25.1%)	
1	725 (34.2%)	
2	427 (20.1%)	
3+	436 (20.6%)	
Disease Severity	*2120*	
No limiting conditions	1554 (73.3%)	
Presence of a limiting condition	566 (26.7%)	
Medication Data	Age 69	Percentage Change^a^
Medication Use From Specific BNF Chapter		
(1) Gastrointestinal	519 (24.5%)	+ 7.3%
(2) Cardiovascular	1171 (55.2%)	+ 13.4%
(3) Respiratory	268 (12.6%)	+ 1.3%
(4) Central Nervous System	460 (21.7%)	+ 1.6%
(5) Anti-Infective Agents	53 (2.5%)	-0.8%
(6) Endocrine	453 (21.4%)	+ 3.2%
(7) Obs, Gynae & Urinary	148 (7.0%)	+ 2.8%
(8) Malignancy & Immune	44 (2.1%)	+ 1.0%
(9) Nutrition & Blood	241 (11.2%)	+ 1.3%
(10) Musculoskeletal	194 (9.1%)	-3.1%
(11) Ophthalmic	95 (4.5%)	+ 1.6%
(12) Ear, Nose & Throat	60 (2.8%)	- 0.9%
(13) Dermatology	61 (2.9%)	- 0.4%
Number of Medications Used		
0	430 (20.3%)	- 8.6%
1 to 4	1209 (57.0%)	+ 5.5%
5 to 8 (Polypharmacy)	384 (18.1%)	+ 2.3%
9+ (Excessive Polypharmacy)	99 (4.7%)	+ 0.8%
Cardiological Polypharmacy		
None	951 (44.8%)	- 13.4%
1 (Monotherapy)	379 (17.9%)	+ 4.0%
2 to 4 (Polypharmacy)	684 (32.2%)	+ 9.2%
5+ (Extreme Polypharmacy)	108 (5.1%)	+ 0.2%
Non-Cardiological Polypharmacy		
None	802 (37.8%)	- 4.5%
1 (Monotherapy)	505 (23.8%)	+ 0.6%
2 to 4 (Polypharmacy)	643 (30.3%)	+ 2.7%
5+ (Extreme Polypharmacy)	172 (8.1%)	+ 1.2%

^a^ Percentage change between overall prevalence of medication category between data collections at age 60–64 and 69, using same sample of 2001 individuals with data at both time points

### Medication use at age 69

**Fig. 1:**
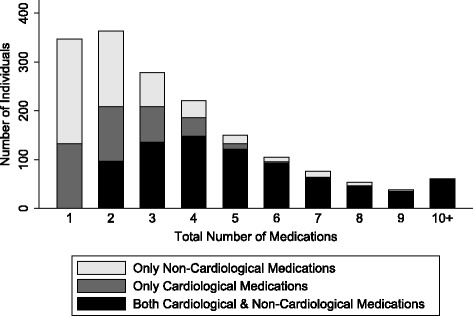
Composition of medications at age 69

At age 69, 79.7% were taking one or more medications; the maximum number taken was 16 and the median number was 2 (Fig. 1). Over half (55.2%) were taking medications for cardiovascular disorders (Table 1). The other common BNF categories of medication were for: gastrointestinal disorders (mainly gastro-oesophageal reflux disease and laxatives) taken by 24.5%; disorders of the central nervous system (mainly analgesics and antidepressants) taken by 21.7%; and endocrine disorders (mainly diabetes and hypothyroidism) taken by 21.3%. One in 20 (4.7%) were taking 9 or more medications and a fifth (18.1%) were taking between 5 and 8.

For most BNF categories, users were prescribed a single medication only. However for cardiological polypharmacy, the use of multiple co-existing agents was common. A single agent was used by less than a fifth (17.9%) of individuals, a third (32.2%) were on two to four, and one in twenty (5.1%) was prescribed over five cardiological medications. When accounting only for all non-cardiological medications collectively, a single medication was taken by 37.8% of the sample, two to four medications by 23.8% and over five medications by 8.1% (Fig. 1).

### Change in medication use since age 60–64

Of the 2122, 1981 (93.3%) participants also had medication data at ages 60–64. Total medication use increased with age for the majority of participants. Fewer individuals remained free from any medication use, falling from 29.2% at ages 60–64 to 20.6% at age 69. Likewise, the number of medications taken by each individual also increased. The greatest proportional increase was among those taking between 1 and 4 medications (+ 5.5%), though there was also an increase in numbers of individuals taking 5 to 8 medications (+ 2.3%) and over 9 medications (+ 0.8%) (Table 1). Cardiological polypharmacy also increased, with the proportion of individuals on two to four cardiological medications showing the largest increase, rising by almost one in ten (9.2%).

Alongside this increase in the number of medications, the composition of medication types taken also altered among the study population. There were increases in medication use from most BNF chapter groups, with the greatest increases in the proportion of cardiovascular (+ 13.4%) and gastrointestinal (+ 7.3%) medications. A few medication subgroups decreased by a small proportion with advancing age, for example musculoskeletal medication (− 3.1%), largely due to a reduction in non-steroidal anti-inflammatory prescriptions.

### Variation in polypharmacy by gender, socioeconomic factors and disease burden

Of the 2122 participants with valid medication data, 2001 (94.3%) also had data available for educational attainment by age 26, social class by age 53, total number of chronic diseases at age 69, and the presence of functional limitation due to disease.

There were few gender differences in total polypharmacy prescription; there were more pronounced differences by education, social class, number of diagnosed diseases and disease severity (Table 2). Not surprisingly, the number of diagnosed diseases was a major risk for all types of medication use; as was disease severity. In gender-adjusted models, higher education was associated with lower likelihood of any medication, polypharmacy and extreme polypharmacy. For example the relative risk ratio (RRR) for extreme polypharmacy was 0.2, 95% confidence intervals (CI) 0.1–0.5 for those with advanced secondary qualifications, and 0.6 (95% CI 0.3–1.1) for those with secondary level qualifications compared with those with no qualifications (*p* < 0.001). These estimates and those for polypharmacy and any medications were partly attenuated on adjusting first for social class and then disease burden, but an inverse association between advanced secondary qualifications and total polypharmacy remained. The inverse association between non-manual social class and total polypharmacy was weaker and more attenuated by education and disease burden.

**Table 2:** Relative Risk Ratios for total polypharmacy by gender, socioeconomic factors and disease burden

	Gender Adjusted Relative Risk Ratios				Model One				Model Two			
	RRR	95% LCI	95% UCI	*p*	RRR	95% LCI	95% UCI	*p*	RRR	95% LCI	95% UCI	*p*
No Medication	*(reference)*				*(reference)*				*(reference)*			
No Polypharmacy (1–4 Meds)												
Gender												
Male	*(reference)*			0.870	*(reference)*			0.496	*(reference)*			0.125
Female	1.0	0.8	1.2		0.9	0.7	1.2		0.8	0.6	1.1	
Educational Qualifications by Age 26												
None	*(reference)*			0.011	*(reference)*			0.010	*(reference)*			0.017
Vocational or O-Level Level	1.0	0.7	1.3		1.0	0.7	1.3		1.0	0.7	1.4	
A-Level or Higher	0.7	0.5	0.9		0.7	0.5	0.9		0.7	0.5	0.9	
Social Class (age 53)												
Manual	*(reference)*			0.624	*(reference)*			0.592	*(reference)*			0.498
Non-Manual	0.9	0.7	1.2		1.1	0.8	1.5		1.1	0.8	1.5	
Number of Diagnosed Diseases												
None	*(reference)*			< 0.001					*(reference)*			< 0.001
One	3.2	2.5	4.2						3.1	2.4	4.1	
Two	6.9	4.6	10.2						6.5	4.3	9.6	
Three or More	9.9	5.7	17.4						8.9	5.1	15.7	
Disease Severity												
No Limiting Conditions	*(reference)*			< 0.001					*(reference)*			< 0.001
Limiting Condition	2.7	1.9	3.9						2.1	1.4	3.0	
Polypharmacy (5–8 Meds)												
Gender												
Male	*(reference)*			0.937	*(reference)*			0.595	*(reference)*			0.044
Female	1.0	0.7	1.3		0.9	0.7	1.2		0.7	0.5	1.0	
Educational Qualifications by Age 26												
None	*(reference)*			< 0.001	*(reference)*			< 0.001	*(reference)*			< 0.001
Vocational or O-Level Level	0.8	0.5	1.0		0.7	0.5	1.1		0.7	0.5	1.2	
A-Level or Higher	0.3	0.2	0.5		0.4	0.2	0.5		0.3	0.2	0.5	
Social Class (age 53)												
Manual	*(reference)*			0.001	*(reference)*			0.290	*(reference)*			0.853
Non-Manual	0.6	0.4	0.8		0.8	0.6	1.2		1.0	0.6	1.4	
Number of Diagnosed Diseases												
None	*(reference)*			< 0.001					*(reference)*			< 0.001
One	6.6	4.0	10.8						5.8	3.5	9.5	
Two	22.0	12.4	39.0						18.1	10.1	32.3	
Three or More	93.0	47.4	82.3						67.0	33.8	132.9	
Disease Severity												
No Limiting Conditions	*(reference)*			< 0.001					*(reference)*			< 0.001
Limiting Condition	7.7	5.2	11.4						4.6	3.0	7.0	
Extreme Polypharmacy (9 + Meds)												
Gender												
Male	*(reference)*			0.496	*(reference)*			0.499	*(reference)*			0.072
Female	0.9	0.5	1.3		0.9	0.5	1.4		0.6	0.4	1.0	
Educational Qualifications by Age 26												
None	*(reference)*			< 0.001	*(reference)*			< 0.001	*(reference)*			< 0.001
Vocational or O-Level Level	0.5	0.3	0.8		0.5	0.3	1.0		0.6	0.3	1.1	
A-Level or Higher	0.2	0.1	0.4		0.3	0.1	0.5		0.2	0.1	0.5	
Social Class (age 53)												
Manual	*(reference)*			< 0.001	*(reference)*			0.046	*(reference)*			0.390
Non-Manual	0.4	0.2	0.6		0.6	0.4	1.0		0.8	0.4	1.4	
Number of Diagnosed Diseases												
None	*(reference)*			< 0.001					*(reference)*			< 0.001
One	2.1	0.6	7.0						1.6	0.5	5.6	
Two	19.0	6.5	55.7						13.6	4.5	40.5	
Three or More	201.9	70.7	576.7						109.9	37.6	321.1	
Disease Severity												
No Limiting Conditions	*(reference)*			< 0.001					*(reference)*			< 0.001
Limiting Condition	21.8	12.6	38.0						10.6	5.9	19.3	

Model One: Gender, Education & Social Class. Model Two: Gender, Education, Social Class, Disease Burden & Severity Risk ratios rounded to a single decimal place

Cardiological polypharmacy, unlike total polypharmacy, varied by gender. Women were less likely to have cardiological polypharmacy (RRR 0.5, 95% CI 0.4–0.6, *p* < 0.001) and extreme cardiological polypharmacy (RRR 0.3, 95% CI 0.2–0.4, *p* < 0.001) (Table 3). Higher levels of education were inversely associated with all medication use in gender adjusted models, before and after adjusting for social class and then disease burden. The estimates for non-manual social class were attenuated by education and by disease burden. Diagnosed diseases again proved a major driver of cardiological polypharmacy, though disease severity was not associated with cardiological polypharmacy in fully adjusted models.

**Table 3:** Relative Risk Ratios for cardiological polypharmacy by gender, socioeconomic factors and disease burden

	Gender Adjusted Relative Risk Ratios				Model One				Model Two			
	RRR	95% LCI	95% UCI	*p*	RRR	95% LCI	95% UCI	*p*	RRR	95% LCI	95% UCI	*p*
No Medication	*(reference)*				*(reference)*				*(reference)*			
Monotherapy (1 Med)												
Gender												
Male	*(reference)*			0.303	*(reference)*			0.245	*(reference)*			0.043
Female	0.9	0.7	1.1		0.9	0.7	1.1		0.8	0.6	1.0	
Educational Qualifications by Age 26												
None	*(reference)*			0.008	*(reference)*			0.003	*(reference)*			0.004
Vocational or O-Level Level	0.6	0.5	0.9		0.6	0.4	0.8		0.6	0.4	0.8	
A-Level or Higher	0.7	0.5	0.9		0.6	0.4	0.8		0.6	0.4	0.8	
Social Class (age 53)												
Manual	*(reference)*			0.933	*(reference)*			0.189	*(reference)*			0.116
Non-Manual	1.0	0.8	1.3		1.2	0.9	1.7		1.3	0.9	1.8	
Number of Diagnosed Diseases												
None	*(reference)*			< 0.001					*(reference)*			< 0.001
One	1.7	1.2	2.3						1.7	1.3	2.4	
Two	1.9	1.3	2.8						2.0	1.4	3.0	
Three or More	4.9	3.2	7.4			0.9	1.7		5.4	3.5	8.3	
Disease Severity												
No Limiting Conditions	*(reference)*			0.950					*(reference)*			0.090
Limiting Condition	1.0	0.7	1.4						0.8	0.6	1.0	
Polypharmacy (2–4 Meds)												
Gender												
Male	*(reference)*			< 0.001	*(reference)*			< 0.001	*(reference)*			< 0.001
Female	0.7	0.5	0.8		0.6	0.5	0.8		0.5	0.4	0.6	
Educational Qualifications by Age 26												
None	*(reference)*			< 0.001	*(reference)*				*(reference)*			< 0.001
Vocational or O-Level Level	0.7	0.5	0.9		0.7	0.5	0.9	< 0.001	0.7	0.5	1.0	
A-Level or Higher	0.5	0.4	0.7		0.5	0.4	0.7		0.5	0.4	0.7	
Social Class (age 53)												
Manual	*(reference)*			0.027	*(reference)*			0.880	*(reference)*			0.626
Non-Manual	0.8	0.6	1.0		1.0	0.8	1.3		1.1	0.8	1.4	
Number of Diagnosed Diseases												
None	*(reference)*			< 0.001					*(reference)*			< 0.001
One	7.4	5.0	10.8						7.2	4.9	10.6	
Two	13.4	8.8	20.2						13.3	8.8	20.2	
Three or More	40.2	25.6	63.2						39.5	24.8	62.3	
Disease Severity												
No Limiting Conditions	*(reference)*			< 0.001					*(reference)*			0.923
Limiting Condition	1.8	1.5	2.3						1.0	0.8	1.3	
Extreme Polypharmacy (5+ Meds)												
Gender												
Male	*(reference)*			< 0.001	*(reference)*			< 0.001	*(reference)*			< 0.001
Female	0.4	0.2	0.6		0.4	0.2	0.6		0.3	0.2	0.4	
Educational Qualifications by Age 26												
None	*(reference)*			< 0.001	*(reference)*			< 0.001	*(reference)*			< 0.001
Vocational or O-Level Level	0.4	0.2	0.6		0.4	0.2	0.7		0.4	0.2	0.8	
A-Level or Higher	0.3	0.2	0.4		0.3	0.2	0.5		0.3	0.2	0.5	
Social Class (age 53)												
Manual	*(reference)*			0.001	*(reference)*			0.305	*(reference)*			0.852
Non-Manual	0.5	0.3	0.7		0.8	0.5	1.3		1.0	0.6	1.6	
Number of Diagnosed Diseases												
None	*(reference)*			< 0.001					*(reference)*			< 0.001
One	3.2	1.1	9.2						3.0	1.0	8.6	
Two	13.6	5.0	36.6						11.8	4.3	32.2	
Three or More	86.5	33.1	226.3						66.8	25.0	178.5	
Disease Severity												
No Limiting Conditions	*(reference)*			< 0.001					*(reference)*			0.056
Limiting Condition	4.0	2.6	6.2						1.6	1.0	2.6	

Model One: Gender, Education & Social Class. Model Two: Gender, Education, Social Class, Disease Burden & Severity

Risk ratios rounded to a single decimal place

Patterns for non-cardiological polypharmacy contrasted with those for cardiological polypharmacy, with women having an increased risk of non-cardiological monotherapy, non-cardiological polypharmacy, and over double the risk of extreme non-cardiological polypharmacy (RRR 2.2 95% CI 1.5–3.3, *p* < 0.001) (Table 4). The inverse associations between higher levels of education and polypharmacy and extreme polypharmacy were again observed, even in the fully adjusted models, although to a lesser degree than for cardiological polypharmacy. Non-manual social class initially showed an inverse association in gender adjusted models, but this was again explained by education. The number of doctor diagnosed diseases and disease severity remained drivers of non-cardiological polypharmacy, although the association with the number of diagnosed diseases was not as pronounced as for cardiological medications.

**Table 4:** Relative Risk Ratios for non-cardiological polypharmacy by gender, socioeconomic factors and disease burden

	Gender Adjusted Relative Risk Ratios				Model One				Model Two			
	RRR	95% LCI	95% UCI	p	RRR	95% LCI	95% UCI	p	RRR	95% LCI	95% UCI	p
No Medication	*(reference)*				*(reference)*				*(reference)*			
Monotherapy (1 Med)												
Gender												
Male	*(reference)*			0.013	*(reference)*			0.018	*(reference)*			0.034
Female	1.3	1.1	1.7		1.3	1.1	1.7		1.3	1.0	1.6	
Educational Qualifications by Age 26												
None	*(reference)*			0.123	*(reference)*			0.325	*(reference)*			0.433
Vocational or O-Level Level	1.0	0.7	1.3		1.0	0.7	1.4		1.0	0.7	1.4	
A-Level or Higher	0.8	0.6	1.0		0.8	0.6	1.1		0.9	0.6	1.2	
Social Class (age 53)												
Manual	*(reference)*			0.056	*(reference)*			0.201	*(reference)*			0.302
Non-Manual	0.8	0.6	1.0		0.8	0.6	1.1		0.9	0.6	1.1	
Number of Diagnosed Diseases												
None	*(reference)*			< 0.001					*(reference* ***)***			< 0.001
One	1.6	1.2	2.1						1.5	1.1	2.0	
Two	2.4	0.7	3.3						2.2	1.5	3.1	
Three or More	2.3	1.6	3.4						2.0	1.3	2.9	
Disease Severity												
No Limiting Conditions	*(reference)*			< 0.001					*(reference)*			< 0.001
Limiting Condition	2.3	1.7	3.1						2.0	1.5	2.7	
Polypharmacy (2–4 Meds)												
Gender												
Male	*(reference)*			< 0.001	*(reference)*			< 0.001	*(reference)*			< 0.001
Female	1.7	1.4	2.1		1.7	1.3	2.1		1.6	1.3	2.0	
Educational Qualifications by Age 26												
None	*(reference)*			< 0.001	*(reference)*			0.001	*(reference)*			0.005
Vocational or O-Level Level	0.8	0.6	1.1		0.9	0.6	1.1		0.9	0.6	1.2	
A-Level or Higher	0.6	0.5	0.8		0.6	0.5	0.8		0.6	0.5	0.8	
Social Class (age 53)												
Manual	*(reference)*			0.038	*(reference)*			0.593	*(reference)*			0.885
Non-Manual	0.8	0.6	1.0		0.9	0.7	1.2		1.0	0.8	1.4	
Number of Diagnosed Diseases												
None	*(reference)*			< 0.001					*(reference)*			< 0.001
One	2.2	1.6	3.0						2.0	1.5	2.7	
Two	3.9	2.8	5.6						3.4	2.4	4.8	
Three or More	6.5	4.5	9.3						4.8	3.3	7.0	
Disease Severity												
No Limiting Conditions	*(reference)*			< 0.001					*(reference)*			< 0.001
Limiting Condition	3.9	3.0	5.2						3.0	2.3	4.0	
Extreme Polypharmacy (5+ Meds)												
Gender												
Male	*(reference)*			< 0.001	*(reference)*			< 0.001	*(reference)*			< 0.001
Female	2.2	1.6	3.1		2.2	1.5	3.1		2.2	1.5	3.3	
Educational Qualifications by Age 26												
None	*(reference)*			< 0.001	*(reference)*			< 0.001	*(reference)*			< 0.001
Vocational or O-Level Level	0.6	0.4	0.9		0.7	0.4	1.0		0.7	0.4	1.1	
A-Level or Higher	0.3	0.2	0.4		0.3	0.2	0.5		0.3	0.2	0.5	
Social Class (age 53)												
Manual	*(reference)*			< 0.001	*(reference)*			0.102	*(reference)*			0.791
Non-Manual	0.5	0.3	0.7		0.7	0.5	1.1		0.9	0.6	1.5	
Number of Diagnosed Diseases												
None	*(reference)*			< 0.001					*(reference)*			< 0.001
One	3.0	1.5	6.0						2.4	1.1	4.9	
Two	6.6	3.2	13.7						4.6	2.2	9.6	
Three or More	29.5	15.0	57.9						15.1	7.5	30.4	
Disease Severity												
No Limiting Conditions	*(reference)*			< 0.001					*(reference)*			< 0.001
Limiting Condition	14.2	9.6	21.0						9.0	6.0	13.6	

Model One: Gender, Education & Social Class. Model Two: Gender, Education, Social Class, Disease Burden & Severity

Risk ratios rounded to a single decimal place

## Discussion

At age 69, total polypharmacy was present in over a fifth of individuals in a nationally representative British cohort, and over half of this sample received at least one cardiovascular prescription. The prevalence of medication use increased with age across the seventh decade, with the greatest increase seen in the volume of cardiovascular prescriptions, particularly in those prescribed between two to four cardiological medications. Disease burden was a major predictor of all types of polypharmacy. Those with higher education were less likely to be prescribed polypharmacy, even when taking this burden into account, along with gender and social class. The associations with education were stronger than those with social class, and all types of polypharmacy were mediated by level of education. Gender differences were more varied; no differences in total polypharmacy were apparent at age 69, yet women were less likely than men to have cardiovascular prescriptions, and more likely to have non-cardiovascular medications.

The primary strengths of this study are the nature of the NSHD being representative of the general population, and having age homogeneity. The measures available allowed accurate capture of a potentially at-risk group for polypharmacy and patterns of sociodemographic and health related factors and disease burden on pharmaceutical prescriptions to be estimated. In addition, the longitudinal data allow for description of change in prescribed medications. One limitation is that medication data relied on self-reports, albeit collected by research nurses, and making use of prescription lists. Evidence suggests that our method should provide robust medication data, as self-reported measures alone correlate well with pharmacy prescription records; identifying most regular medications and only occasionally missing ‘as required’ and non-oral medications, such as transdermal patches [28]. The accuracy of self-reported diagnosed disease varies dependent on condition severity [29], though most major diseases have fairly high accuracy using this measure. Prior work on NSHD showed self-reported diabetes diagnoses were comparable with GP records in 95% of cases [30]. Additionally, capping our measure at 3+ diseases reduces the potential for variance at higher levels of disease burden. Similarly, our measure of disease severity, defined as whether long-term illness limits activity, is likely to detect most severe diseases within the sample population – although it may be influenced by participant perception of expected function. No data were available to account for the primary general practitioner’s (GPs) prescribing habits, which holds significant associations with major polypharmacy [31]. Given the geographical spread of study members across England, Scotland and Wales, a high variation in GP prescribing patterns is likely to exist, but this is unlikely to alter results. Also of note, data used here were collected at two time points, over 5 years apart. Participants may have taken numerous prescriptions of a shorter duration between these time points. This is suggested, for example, by the low numbers of anti-infective prescriptions among the cohort. However, given that it is more important to understand chronicity of medication use in polypharmacy, this is unlikely to affect our findings.

The overall prevalence of polypharmacy within the study sample correlates well with that of a large scale health records study in Scotland [2], with similar proportions of individuals taking over five medications, and a high prevalence of cardiological medications. The increase in the number of medications taken by participants between the ages of 60–64 and 69 supports prior evidence suggesting that medication use increases with age [2, 11, 32]; of note here is the considerable increase in cardiovascular prescriptions revealed by this study. Epidemiological evidence from a Swedish cohort study suggested that women were 50% more likely to be medicated at any age, though the prevalence of polypharmacy (over five medications) was roughly equivalent for both genders by age seventy [11]. A large increase in male polypharmacy between the ages of 60 and 69 has previously been noted in large population databases, and appears to have been replicated in NSHD [33]. Total polypharmacy in this study follows this trend; however, gender appears to play a role in the composition of that polypharmacy, with men prescribed more cardiovascular drugs than women, in keeping with prior studies on cardiological polypharmacy [20, 21], even after additionally adjusting for disease burden in the current study.

Our findings for multi-morbidity, represented here by the number of diagnosed diseases, supports prior evidence that this is the major driver of polypharmacy [3, 11, 15], although again, this appears to have a greater impact on the number of cardiovascular rather than non-cardiovascular medications. Disease severity however was more strongly associated with non-cardiological polypharmacy, possibly accounting for the greater breadth of non-cardiological diseases in the general population leading to more variation in their severity; with more severe diseases requiring more medication to treat. Low levels of education have previously been highlighted as a predictor of polypharmacy [13, 32, 34] but only some studies have controlled for multi-morbidity [14, 34]. In our study, these associations persisted even when adjusting for disease burden, and were found to be more pronounced for cardiovascular medications than for non-cardiovascular medications. Cardiovascular disorders are strongly associated with negative health behaviours, such as smoking and reduced exercise [35], and these negative health behaviours are in turn associated with lower levels of education and social class [36, 37]. The resulting disorders such as myocardial infarction, ischaemic heart disease and heart failure, commonly warrant multiple pharmacological agents for treatment [27]; leading to a high volume of cardiovascular polypharmacy in this group. Yet this may not entirely explain this phenomenon, as we found that these associations with education also existed for non-cardiological polypharmacy, and persisted when adjusting for disease severity.

The reasons for the inverse associations between education and polypharmacy are therefore potentially multifaceted, and could indicate an additional disparity in interactions with healthcare services. Individuals with higher levels of education may be more likely to view encounters with medical professionals as a shared responsibility, and to question treatment options [38, 39]. Therefore less educated individuals may accept or be offered pharmacological treatment more readily than those who have spent time exploring side-effect profiles and alternative therapies. Indeed, prior analysis of GP data suggests that more money is spent on prescriptions for individuals of lower socioeconomic position [40]. Further research on consultation practice and prescriptions would be warranted to explore this trend.

## Conclusions

When defining polypharmacy by quantity of drugs alone, key differences in the composition of the medications involved are neglected. Men and those with lower levels of education take more cardiovascular drugs, which have a higher risk of potentially adverse drug-drug interactions, and may have later life consequences such as increased mortality or reduced functional outcomes. Future research on polypharmacy outcomes should aim to account for variations in both the prevalence and composition of polypharmacy by gender and education as well as disease burden. While further work is required to unpick these associations, they highlight the potential for targeted medication reviews in high risk populations.

## Abbreviations

BNF: British national formulary
LCI: Lower confidence interval
MRC: Medical research council
NSHD: National survey for health and development
RRR: Relative risk ratio
UCI: Upper confidence interval
